# Insulin resistance (HOMA-IR) cut-off values and the metabolic syndrome in a general adult population: effect of gender and age: EPIRCE cross-sectional study

**DOI:** 10.1186/1472-6823-13-47

**Published:** 2013-10-16

**Authors:** Pilar Gayoso-Diz, Alfonso Otero-González, María Xosé Rodriguez-Alvarez, Francisco Gude, Fernando García, Angel De Francisco, Arturo González Quintela

**Affiliations:** 1 Clinical Epidemiology Unit, Hospital Clínico Universitario, A Choupana, s/n, 15706, Santiago de Compostela, Spain; 2 Instituto de Investigación Sanitaria de Santiago (IDIS), Santiago de Compostela, Spain; 3 Nephrology Department, C. H.U. de Ourense, Ourense, Spain; 4 Clinical Epidemiology Unit, Puerta de Hierro University Hospital, Madrid, Spain; 5 Nephrology Department, Hospital Marques de Valdecilla, Santander, Spain

**Keywords:** Insulin resistance, Gender, Cardio metabolic risk, Metabolic syndrome

## Abstract

**Background:**

Insulin resistance has been associated with metabolic and hemodynamic alterations and higher cardio metabolic risk. There is great variability in the threshold homeostasis model assessment of insulin resistance (HOMA-IR) levels to define insulin resistance. The purpose of this study was to describe the influence of age and gender in the estimation of HOMA-IR optimal cut-off values to identify subjects with higher cardio metabolic risk in a general adult population.

**Methods:**

It included 2459 adults (range 20–92 years, 58.4% women) in a random Spanish population sample. As an accurate indicator of cardio metabolic risk, Metabolic Syndrome (MetS), both by International Diabetes Federation criteria and by Adult Treatment Panel III criteria, were used. The effect of age was analyzed in individuals with and without diabetes mellitus separately. ROC regression methodology was used to evaluate the effect of age on HOMA-IR performance in classifying cardio metabolic risk.

**Results:**

In Spanish population the threshold value of HOMA-IR drops from 3.46 using 90th percentile criteria to 2.05 taking into account of MetS components. In non-diabetic women, but no in men, we found a significant non-linear effect of age on the accuracy of HOMA-IR. In non-diabetic men, the cut-off values were 1.85. All values are between 70th-75th percentiles of HOMA-IR levels in adult Spanish population.

**Conclusions:**

The consideration of the cardio metabolic risk to establish the cut-off points of HOMA-IR, to define insulin resistance instead of using a percentile of the population distribution, would increase its clinical utility in identifying those patients in whom the presence of multiple metabolic risk factors imparts an increased metabolic and cardiovascular risk. The threshold levels must be modified by age in non-diabetic women.

## Background

Insulin resistance (IR) is a feature of disorders such as diabetes mellitus type 2 (DM2) and is also implicated in obesity, hypertension, cancer or autoimmune diseases [1-3]. Insulin resistance (IR) has been proposed, more than a primary cause, as a sort of final common pathway for negative environmental factors, which interact with the individual genetic background to cause metabolic and hemodynamic alterations and is associated with inflammation [4,5].

Metabolic syndrome (MetS) definition is widely used as a practical tool to describe a cluster of clinical signs (central obesity, dyslipidemia, impaired glucose metabolism, and elevated blood pressure) that regardless of cause, identifies individuals at risk of atherosclerotic cardiovascular disease (CVD), and DM2 [6-9]. The worldwide prevalence of these factors has risen dramatically in recent decades [10-12].

The Homeostasis Model Assessment of IR (HOMA-IR) has proved to be a robust tool for the surrogate assessment of IR [13,14]. However, there is great variability in the threshold HOMA-IR levels to define IR. Population based studies for defining cut-off values of HOMA-IR for the diagnosis of IR had been conducted in different geographic areas [15-22]. Table 1 shows the cut-off values, as can be seen in most of cases the cutoff point’s determination were made on the percentile criterion (80 or 90 according to studies) of values in the general population. However, no studies have examined the ability of proposed cutoff points to identify risk of clinically relevant outcomes [14]. In addition, in these studies the results have been reported without taking into account the possible effects of covariates on test results. However, it is well known that a biomarker’s performance and, by extension, its discriminatory capacity can be affected by covariates [23].

**Table 1:** Summary of reports (sorted by sample size) on HOMA-IR cut-off in different populations

**Study**	**Characteristics of study population**	**Threshold value**	**Criteria**
Hedblad, 2000 [15]	N = 4,816 Sweden, population-based sample	≥ 2.0	75th percentile
Summer, 2008 [16]	N = 2804, U.S. NHANES population, age ≥ 20 yr., normal BMI and fasting glucose	≥2.73	66th percentile
Geloneze, 2006 [17]	N = 1317 Brazilian, age: 40 ± 12 yr, BMI: 34 ± 10 kg/m^2^	≥ 2.77	90th percentile
Esteghamati, 2009 [18]	N = 1,276 Iranian,	≥1.80	ROC
	Age: 38 ± 12 yr, non-diabetic, normotensive	≥1.95	ROC
	IDF-MetS	≥1.6	75th percentile
	ATPIII-MetS	≥1.8	80th percentile
		≥ 2.3	90th percentile
Marques-Vidal, 2002 [19]	N = 1153, France, age: 35–64 yr, population based sample	≥3.8	75th percentile
Do, 2010 [20]	N = 738 Thailand, age: ≥35 yr, normal BMI and fasting glucose	1.55	90th percentile
Miccoli, 2005 [38]	N = 225 Italian, age: 40–79 yr, healthy subjects	≥ 2.77	80th percentile
Nakai, 2002 [22]	N = 161 Japanese, age: 41.6 ± 0.4 yr, healthy subjects	≥ 1.7	90th percentile
Ascaso, 2001 [39]	N = 140 Spanish, age: 7–16 yr	3	ROC
Tome, 2009 [40]	N = 2860 Spanish, population based age: 18–104 yr, BMI: 26.2 ± 4.9 kg/m^2^	2	ROC

In a previous study we showed that there are age and gender-specific differences in HOMA-IR levels, with increased levels in women over fifty years of age [24]. On the other hand, the prevalence of cardio metabolic diseases such as diabetes or central obesity rises with age and shows gender differences [11,12]. All these results suggest the possible effects of both age and gender on the accuracy of HOMA-IR to identify individuals with cardio metabolic risk.

The purpose of the present population-based study was to evaluate the change in defining cut-off values of HOMA-IR for the diagnosis of IR when cardio metabolic risk factors were considered. We currently assess the influence of age and gender on the performance of HOMA-IR levels to identify cardio metabolic risk in an adult population, to better understand the relationship between insulin resistance and cardio metabolic risk.

## Methods

### Setting

The present study was a secondary analysis of data from a survey of the Spanish general adult population (EPIRCE) [25,26]. The EPIRCE is an observational, cross-sectional study that included a randomly selected sample of Spanish persons aged 20 years and older stratified by age, gender, and habitat. The study was primarily intended to investigate the prevalence of chronic kidney disease (CKD) in the Spanish adult population. Details of the study design were previously published [26].

For the present study, data analysis could not be performed in 249 individuals (9.1%) because of a lack of insulin level recording and in 38 (1.4%) individuals because of a lack of waist circumference recording. There were no statistically significant differences between individuals with or without missing data regarding age, gender, hypertension, alcohol intake, or physical activity. Finally, 2459 individuals were selected for study inclusion. People with diabetes (247, 10.0%), defined as a fasting plasma glucose ≥ 7.0 mmol l^-1^ and/or the current use of diabetes medications (32, 1.3%), were included. The average age was 49.4 ± 16.2 years (range 20–92 years). A total of 1436 (58.4%) were women. All participants were Caucasians.

### Anthropometric and clinical measurements

Subjects were considered to have hypertension if they had a mean systolic blood pressure (SBP) ≥140 mmHg and/or diastolic blood pressure (DBP) ≥90 mmHg or used antihypertensive medications.

Waist circumference and body weight and height were measured according to a standard protocol. To measure the waist circumference all researchers followed these instructions: Locate the top of the hip bone (iliac crest) and take the measurement just above this bony landmark, just where one finger can fit between the iliac crest and the lowest rib. Ensure that the tape measure is positioned horizontally, parallel to the floor. Measuring at a level just above the iliac crest, and positioning the tape horizontally, irrespective of whether the umbilicus is above or below the tape, provides the correct waist circumference measurement and should correspond to the maximal abdominal diameter. Ensure that the patient is standing erect and has relaxed the abdominal muscles. Measurement must be taken at the end of normal expiration. The body mass index (BMI) was calculated as the weight (kg) divided by the square of the height (meters).

### Specific laboratory determinations

A blood sample was collected after an overnight fast of >8 h. Plasma glucose levels were measured using a hexokinase enzymatic reference method. Fasting insulin levels were measured using a radioimmunoassay (RIA) method (Coat A Count Insulin, Los Angeles, USA). Fasting lipids were analyzed, and for the present study serum levels of cholesterol ≥5.172 mmol l^-1^ and triglycerides ≥1.7 mmol l^-1^ were considered abnormal.

HOMA-IR was used to evaluate insulin resistance (fasting serum insulin (μU/ml) × fasting plasma glucose (mmol l^-1^)/22.5) [27].

### Definition of metabolic syndrome

As an accurate indicator of cardio metabolic risk, MetS, both by the International Diabetes Federation (IDF) criteria and by the Adult Treatment Panel III (ATP III) criteria, were used. Under the IDF criteria, MetS (MetS_IDF_) was defined as the presence of central obesity (waist circumference ≥94 cm for men and ≥80 cm for women) plus any two of the following risk factors: HDL-cholesterol <1.03 mmol l^-1^ (males) and <1.29 mmol l^-1^ (females) or specific treatment for this lipid abnormality; systolic blood pressure ≥130 or diastolic blood pressure ≥85 mm Hg, or treatment of previously diagnosed hypertension; fasting plasma glucose ≥5.6 mmol l^-1^, or previously diagnosed type 2 diabetes; triglycerides ≥1.7 mmol l^-1^ or specific treatment for this lipid abnormality [28]. According to ATPIII criteria, MetS (MetS_ATPIII_) was defined as the presence of three or more of the following: HDL-cholesterol <1.03 mmol l^-1^ (males) and <1.30 mmol l^-1^ (females) or specific treatment for this lipid abnormality; blood pressure ≥130/85 mm Hg or treatment of previously diagnosed hypertension; fasting plasma glucose ≥5.6 mmol l^-1^, or previously diagnosed type 2 diabetes; triglycerides ≥1.7 mmol l^-1^ or specific treatment for this lipid abnormality; waist circumference ≥102 cm for males and ≥88 cm for females [29].

### Statistical analyses

Baseline subject characteristics are expressed as the mean ± SD or as percentages. Cross-tabulation significance levels were based on Pearson’s chi-square test for categorical variables. The Mann–Whitney U-test and Kruskall-Wallis test were employed for comparison of quantitative variables. Although normally distribution of quantitative variables were verified, non parametric test were used because they are less likely than the parametric test to spuriously indicate significance due to the presence of outliers, they are more robust.

To analyze the effect of age on the accuracy of HOMA-IR when predicting the presence of cardio metabolic risk, a novel non-parametric extension [30] of the induced ROC regression methodology [23,31] was used. Since it is well established that HOMA-IR values behave differently according to gender and diabetes status, the analyses were performed separately in men and in women and in diabetic and non-diabetic individuals. We evaluate the significant effect of age on the accuracy of HOMA-IR and P-values were obtained based on 200 bootstrap replications [30].

When the estimated effect of age on the mean of HOMA-IR probed to be linear, and the estimated variances probed to be constant (independent of age), we reanalyzed the data using the semi-parametric induced ROC regression [23,32].

Finally, in addition to the estimated (age-specific) ROC curve, the Area Under the Curve (AUC) and bootstrap-based confidence intervals were obtained, (b = 500 resamples). The (age-specific) threshold values were also computed based on two different criteria: (a) by setting the specificity at 0.7, and (b) by the Youden Index (YI). Insofar as the computation of the YI is concerned, in those situations where a significant effect of age was detected on the accuracy of HOMA-IR, a modification of the usual definition was used, which takes covariates into account.

All statistical analyses were performed using R software, version 2.12.1 [33]. ROC analyses were performed using the packages pROC [34], ROCRegression and npROCRegression. These last two packages can be obtained by contacting MX Rodriguez-Alvarez (mxrodriguez@uvigo.es).

### Ethical considerations

The Galician Ethical Committee for Clinical Research approved the study protocol. All patients provided informed consent.

## Results

Table 2 summarizes anthropometric, clinical, and biochemical characteristics of the study sample. In the overall data set, the MetS prevalence was 15% for MetS_IDF_ (19.2% in men vs. 12.1% in women, P < 0.0001) and 12.7% for MetS_ATPIII_ (14.9% in men vs. 11.1% in women, P = 0.006). In non-diabetic individuals, but not in diabetic individuals, we found significant differences by gender in components of MetS (data no shown). The percentage of men with positive MetS components of higher triglycerides, blood pressure, and glycemia were significantly higher than women (23% vs. 9.6% (P < 0.001), 32% vs. 19% (P < 0.001) and 21% vs. 13% (P < 0.001)). In contrast, women with positive MetS component of larger waist circumference were significantly higher than men (43.6% vs. 29.8%, ATPIII criteria, P < 0.0001 and 52.7% vs. 35.1%, IDF criteria, P < 0.001).

**Table 2:** Anthropometric, clinical, and biochemical characteristics of patient sample: distribution by gender in diabetic (n = 247) and non-diabetic (n = 2212) individuals

	**Women (1308/128)**	**Men (904/119)**	**Total**
Age (years)			
• Non-diabetic	47.6 ± 15.9	48.2 ± 16.0	47.9 ± 15.9
• Diabetic	64.4 ± 10.7	62.4 ± 10.8	63.4 ± 10.7
Waist circumference (cm)			
• Non-diabetic***	86.8 ± 13.2	96.3 ± 11.3	90.6 ± 13.3
• Diabetic*	101.5 ± 13.5	105.0 ± 11.4	103.1 ± 12.5
BMI (kg/m^2^)			
• Non-diabetic***	26.9 ± 5.4	27.8 ± 4.5	27.3 ± 5.1
• Diabetic***	32.2 ± 5.6	29.4 ± 4.4	31.1 ± 5.2
Systolic blood pressure (mmHg)			
• Non-diabetic***	125.4 ± 21.0	135.8 ± 19.0	129.6 ± 20.8
• Diabetic	145.9 ± 21.1	148.6 ± 21.2	147.3 ± 21.1
Diastolic blood pressure (mmHg)			
• Non-diabetic***	76.6 ± 11.0	81.1 ± 11.4	78.4 ± 11.4
• Diabetic	82.1 ± 11.7	82.1 ± 10.7	82.1 ± 11.2
Triglycerides (mmol l^-1^)			
• Non-diabetic***	1.0 ± 0.6	1.3 ± 0.9	1.1 ± 0.7
• Diabetic	1.5 ± 0.8	1.9 ± 1.9	1.7 ± 1.4
HDL-Cholesterol (mmol/L)			
• Non-diabetic***	2.0 ± 0.5	1.7 ± 0.4	1.9 ± 0.5
• Diabetic**	1.7 ± 0.4	1.6 ± 0.4	1.8 ± 0.4
Fasting insulin (U/l)			
• Non-diabetic**	7.7 ± 4.6	8.5 ± 5.2	8.0 ± 4.9
• Diabetic	11.9 ± 6.2	10.9 ± 6.5	11.4 ± 6.3
Fasting plasma glucose (mmol l^-1^)			
• Non-diabetic***	4.9 ± 0.6	5.1 ± 0.6	5.0 ± 0.6
• Diabetic*	7.8 ± 2.4	8.1 ± 2.5	8.0 ± 2.5
HOMA-IR (units)			
• Non-diabetic	1.9 ± 1.0	2.1 ± 1.2	2.0 ± 1.1
• Diabetic*	1.9 ± 1.0	1.7 ± 1.1	1.9 ± 1.1
Metabolic syndrome			
ATPIII**	11.1% (159)	14.9% (152)	12.7% (311)
• Non-diabetic**	7.6% (99)	11.1% (100)	9.0% (199)
• Diabetic	46.9% (60)	43.7% (52)	45.3% (112)
IDF***	12.1% (174)	19.2% (196)	15.0% (370)
• Non-diabetic***	8.7% (114)	14.9% (135)	11.3% (249)
• Diabetic	46.9% (60)	51.3% (61)	49.0% (121)

Data are presented as mean ± standard deviation, or percentages (n). BMI, body mass index; HDL-Cholesterol, high density lipoprotein-Cholesterol; HOMA-IR, homeostasis model assessment of IR; ATPIII, Third Adult Treatment Panel; IDF, International Diabetes Federation.

Contrast of characteristics by gender was done with the follow statistical significance: *p < 0.05, **p < 0.01, ***p < 0.001.

Mean HOMA-IR levels significantly increased with rising number of MetS components from 1.7 (without MetS components) to 5.3 (with 5 components) (P < 0.0001).

### AUC values of HOMA-IR by age, gender and diabetes status

**Figure 1:**
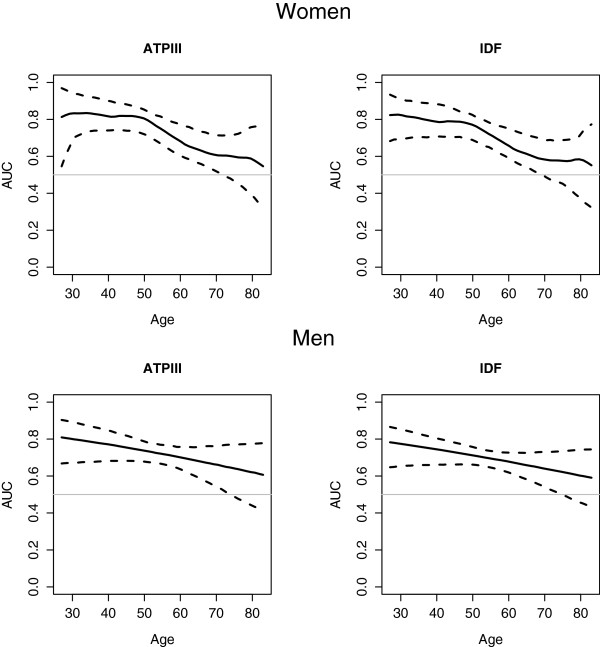
**Performance of HOMA-IR levels for classification of cardio metabolic risk in non-diabetic population.** Influence of age and gender in the area under the ROC curve (AUC), ROC regression models.

The results of the effects of age and gender on the accuracy of HOMA-IR (AUC values) among non-diabetic and diabetic individuals were presented in Table 3. Meanwhile, Figure 1 shows the estimated AUC values by age, with the corresponding 95% point wise bootstrap confidence band in non-diabetic men and women. Regardless of diabetes status, the AUC values of HOMA-IR were slightly higher for MetS_ATPIII_ than MetS_IDF_ (Table 3). The effect of age on the accuracy of HOMA-IR was analyzed in individuals with and without diabetes mellitus separately. As can be seen in Table 3, in non-diabetic women a significant non-linear effect of age on the accuracy of HOMA-IR in identifying MetS, both MetS_ATPIII_ (P = 0.012) and MetS_IDF_ (P < 0.001), was found. The AUC presents a plateau with values greater than 0.7 until 50 years of age. From the age of 50, the AUC decreases progressively. For patients older than 70 years, the bootstrap confidence intervals for the AUC includes 0.5; thus there is no evidence suggesting that HOMA-IR can be used to classify non-diabetic older women with cardio metabolic risk. Table 3 shows the estimated AUC values for ages of 30, 50, and 70 years in our Spanish population. The AUC drops from 0.82 (age 30) to 0.58 (age 70).

**Table 3:** Performance of HOMA-IR values in the classification of cardio metabolic risk (both ATPIII MetS and IDF MetS definition), influence of age and gender

		ROC coefficients*	**P value**	**AUC (95% CI)**
A	Males			
	IDF MetS			
	• Age	0.0102	0.1665	0.69 (0.65, 0.74)
	• Intercept	−1.1411	0.0048	
	ATPIII Mets			
	• Age	0.0117	0.1897	0.72 (0.67, 0.77)
	• Intercept	−1.2976	0.0089	
	Females **			
	IDF MetS			
	• Age 30 yr			0.82 (0.71, 0.90)
	• Age 50 yr		<0.001	0.77 (0.68, 0.82)
	• Age 70 yr			0.58 (0.48, 0.68)
	ATPIII MetS			
	• Age 30 yr			0.83 (0.71, 0.91)
	• Age 50 yr		0.012	0.80 (0.71, 0.85)
	• Age 70 yr			0.61 (0.52, 0.70)
B	Males			
	IDF MetS			
	• Age	−0.0113	0.8998	0.68 (0.59, 0.78)
	• Intercept	0.1406	0.5160	
	ATPIII MetS			
	• Age	−0.0029	0.8595	0.72 (0.62, 0.81)
	• Intercept	−0.4692	0.6515	
	Females			
	IDF MetS			
	• Age	−0.0010	0.9656	0.54 (0.44, 0.64)
	• Intercept	0.0173	0.9914	
	ATPIII MetS			
	• Age	−0.0010	0.9656	0.54 (0.44, 0.64)
	• Intercept	0.0173	0.9914	

Areas under the ROC curves for non-diabetic (A) and diabetic (B) adults (n = 2459).

AUC (95% CI), area under the ROC curve (95% Confidence Interval). *ROC regression models incorporating age as covariate. **The AUC was estimated for three ages (30, 50, and 70 years) to illustrate the performance of HOMA-IR. HOMA-IR, homeostasis model assessment of IR; ATPIII, Third Adult Treatment Panel; IDF, International Diabetes Federation; MetS, Metabolic Syndrome.

However, in non-diabetic men the AUC progressively decreases with age, without statistical significance (P = 0.16, Figure 1). Thus AUC value, 0.69 (0.65, 0.74) for MetS_IDF_ and 0.71 (0.66, 0.76) for MetS_ATPIII_, was estimated without covariates (Table 3).

On the other hand, in diabetic individuals there was no statistically significant effect of age on the accuracy of HOMA-IR. The AUC show an acceptable performance of HOMA-IR in diabetic men, 0.7 (0.6, 0.8), but not in diabetic women 0.54 (0.44, 0.64) (Table 3).

### Cut-off values of HOMA-IR

**Figure 2:**
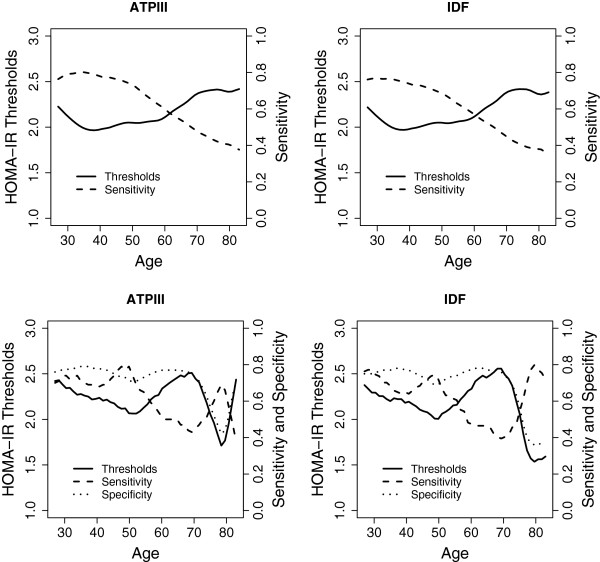
**Optimal HOMA-IR cut point for classification of cardio metabolic risk in non-diabetic women.** The top graphics show the results based on setting the specificity at 0.7, and the bottom graphics the results based on the generalization of the Youden Index. The ATPIII-defined criteria for metabolic syndrome were used on the left, and the IDF-defined criteria for metabolic syndrome on the right.

Table 4 shows gender distribution of HOMA-IR cut-off values, with their corresponding sensitivity and specificity both in diabetic and non-diabetic populations. In non-diabetic individuals we found significantly differences by gender. Figure 2 depicts the estimated HOMA-IR cut-off values by age in non-diabetic women for MetS_ATPIII_ and MetS_IDF_ respectively_._ For MetS_ATPIII_ the optimal HOMA-IR cut-off values ranged from 2.07 (sensitivity, 0.72; specificity, 0.71) at 50 years to 2.47 (sensitivity, 0.44; specificity, 0.74) at 70 years when using YI criteria. Very similar values were found for MetS_IDF_. On the other hand, in non-diabetic men, for MetS_ATPIII_ the optimal HOMA-IR cut-off was 1.85 (sensitivity, 0.78; specificity, 0.57) when YI criteria were used and 2.27 (sensitivity, 0.61) with fixed specificity criteria. Moreover, for MetS_IDF_ the optimal HOMA-IR cut-off was higher, 2.05 (sensitivity, 0.65; specificity, 0.64), when YI criteria were used.

**Table 4:** Gender distribution of HOMA-IR cut-off levels, with their corresponding sensitivity and specificity,for classify of IDF MetS and ATPIII MetS, in diabetic and non-diabetic individuals

**IDF criteria**						
**Population**	**Criterion of specificity = 0.7**			**Youden index criterion**		
	**Cut point**	**Sensitivity**	**Specificity**	**Cut point**	**Sensitivity**	**Specificity**
Diabetic						
Men	1.55	0.60	0.70	1.60	0.59	0.74
Women	2.22	0.37	0.70	1.58	0.68	0.46
Non-diabetic						
Men	2.25	0.57	0.70	2.05	0.65	0.64
Women*						
30 years	2.11	0.77	0.70	2.31	0.71	0.76
50 years	2.05	0.69	0.70	2.05	0.69	0.70
70 years	2.38	0.45	0.70	2.53	0.40	0.75
**ATP III criteria**						
**Population**	**Criterion of specificity = 0.7**			**Youden index criterion**		
	**Cut point**	**Sensitivity**	**Specificity**	**Cut point**	**Sensitivity**	**Specificity**
Diabetic						
Men	1.57	0.64	0.70	1.60	0.63	0.73
Women	2.22	0.37	0.70	1.58	0.68	0.46
Non-diabetic						
Men	2.27	0.61	0.70	1.85	0.78	0.57
Women*						
30 years	2.12	0.79	0.70	2.36	0.73	0.77
50 years	2.05	0.73	0.70	2.07	0.72	0.71
70 years	2.37	0.48	0.70	2.47	0.44	0.74

*In non-diabetic females HOMA-IR cut-off values are estimated for 30, 50, and 70 years of age, because there is a non linear effect of age on test performance to classify IDF-defined MetS (P value < 0.001) and ATP III-defined MetS (P value = 0.012).

HOMA-IR, homeostasis model assessment of IR; ATPIII, Third Adult Treatment Panel; IDF, International Diabetes Federation; MetS, Metabolic Syndrome.

In diabetic individuals the optimal HOMA-IR cut-off value for MetS_ATPIII_ was 1.60 (sensitivity, 0.63; specificity, 0.73) in men and 1.58 (sensitivity, 0.68; specificity, 0.46) in women (YI criteria).

## Discussion

Overall, in non-diabetic individuals the best HOMA-IR cut-off levels ranged from 1.85 in men to 2.07 in women aged 50 years old for the diagnosis of IR take in account cardio metabolic risk. In women without diabetes, the optimal cutoff point should be estimated for each age group due to the non-linear effect of age on the accuracy of HOMA-IR. Even more, in women over 70 years there is no evidence suggesting that HOMA-IR can be used to classify individuals with or without cardio metabolic risk. All values are between the 70th-75th percentiles of HOMA-IR levels in the adult Spanish population [24].

We found lower cut-off values for diabetic than non-diabetic individuals (1.60 vs. 2.05 for MetS_IDF_ in men), probably because in the diabetic population there is an increased prevalence of hypertension, obesity, and dyslipidemia, thus lower HOMA-IR values identifies individuals with three or more MetS components.

In non-diabetic individuals AUC (95%IC) was 0.69 (0.64, 0.74) for MetS_IDF_ and 0.72 (0.67, 0.77) for MetS_ATPIII_ in men and 0.77 (0.68, 0.82) for MetS_IDF_ and 0.80 (0.71, 0.85) for MetS_ATPIII_ in women. These results are similar to the study by Esteghamati that found an AUC of 0.65 (0.63, 0.67) for MetS_IDF_ and 0.68 (0.66, 0.70) for MetS_ATPIII_[35].

There is a significant effect of age on the diagnostic performance of HOMA-IR levels to identify cardio metabolic risk in non-diabetic women; however, there is no evidence of a significant effect in non-diabetic men. Meanwhile, in diabetic individuals we did not find a statistically significant effect of age on the accuracy of HOMA-IR.

The AUC in non-diabetic women presents a plateau, with values greater than 0.7, until patients are in their fifties. Recent studies reported marked gender differences with regard to degrees of IR and body composition [24,26]. The age effect found in non-diabetic women in our study may reflect the effect of menopausal changes (decreased estrogens levels and increased visceral adipose tissue, VAT) on HOMA-IR performance, with a higher utility to identify cardio metabolic risk below age 50.

Insulin resistance increases atherogenesis and atherosclerotic plaque instability by inducing proinflammatory activities on vascular and immune cells [36,37]. HOMA-IR is a robust surrogate method to estimate IR in epidemiologic or clinical setting. However, there is great variability in their threshold levels; as can be seen in Table 1, usually the cut-off values of HOMA-IR were defined by population-based percentiles criteria. Furthermore, these cut-off values are different according to ethnicity, clinical methods of estimation, and metabolic conditions of populations studied [14]. To increase the clinical utility of HOMA-IR values, we study its ability to classify those individuals with multiple metabolic risk factors. In Spanish population the threshold value of HOMA-IR drops from 3.46 using 90th percentile criteria [24] to 2.05 take into account MetS components.

Our HOMA-IR cut-off levels are relatively low compared to those reported in a study of healthy Italian patients [38] with a value of 2.77, and in a Spanish non-diabetic population [39], with a value of 3.8. Both studies used the 80th or 90th percentile as cut-off selection criteria. On the other hand, our values are slightly higher than those reported in an Iranian population-based study with 1.77, using YI as cut-off selection criteria [26], but in this case the value was estimated pooled in men and women.

The prevalence of MetS (15% for IDF and 12.7% for MetS_ATPIII_) was quite similar to that found in northwest Spain (18.3% for MetS_IDF_ and 15.0% for MetS_ATPIII_) [40] and in other European population-based studies [41]. On the other hand, it is significantly lower compared with the NHANES study [42], 23.7%, and SuRFNCD-2007 study [26], 33.6%, probably because of the higher prevalence of obesity and other metabolic alterations in US and eastern Asia compared to the Spanish population [11,12].

The strengths of this study include the use of a large, diverse, and well-characterized population-based sample of adults. We used a novel non-parametric extension of the induced ROC regression methodology to analyze the effect of age on the accuracy of HOMA-IR when predicting the presence of cardio metabolic risk. The induced ROC regression methodology applied in this study is based on first evaluating the effect of covariates on the biomarker in healthy and diseased populations separately, and then computing the covariate effects on the associated ROC curve by deriving the induced form of the ROC curve.

We acknowledge limitations to our approach as well. The cross-sectional nature of our study does not allow us to draw conclusions regarding causality between IR and cardio metabolic risk. Furthermore the small sample size of diabetic patients does not allow us to draw conclusions about the performance of HOMA-IR in identifying cardio metabolic risk in diabetics. More prospective, population-based studies are needed to elucidate these concerns.

## Conclusions

We propose the addition of the components of MetS analysis as a criterion to establish the cut-off points of HOMA-IR to define IR instead of using a percentile of the population distribution. The consideration of the attendant risk of cardiovascular and metabolic diseases to establish this cut-off point would increase its clinical utility in identifying those patients in whom the presence of multiple metabolic risk factors imparts an increased metabolic and cardiovascular risk.

In summary, with the increased prevalence of obesity and diabetes [11,12], the study of IR and body composition has become an important area of research in developed countries and a central public health task.

The effect of age and gender on the ability of HOMA-IR to identify subjects with cardio metabolic risk phenotype should be taken into account in the estimation of their values in different populations. The threshold HOMA-IR levels to define IR must be modified by age in non-diabetic women.

## Competing interests

All authors declare that they have no competing interests.

## Authors’ contributions

PG conceived of the study, and participated in its design and coordination and helped to draft the manuscript. AO participated in the design of the study, have made substantial contributions to acquisition of data and helped to draft the manuscript MXRA performed the statistical analysis and helped to draft the manuscript. FGu, FGa, AF and AG participated in the analysis and interpretation of data and helped to draft the manuscript. All authors read and approved the final manuscript.

## Pre-publication history

The pre-publication history for this paper can be accessed here:

http://www.biomedcentral.com/1472-6823/13/47/prepub
